# Improvement in Survival Rates and Quality of Life Among Patients Surgically Treated for Squamous Cell Carcinoma of the Oral Cavity

**DOI:** 10.1007/s12663-024-02289-z

**Published:** 2024-07-29

**Authors:** Giancarlo Tirelli, Enrico Zanelli, Jerry Polesel, Nicoletta Gardenal, Vittorio Ramella, Chiara Mineo, Simone Zucchini, Marco Piovesana, Vittorio Grill, Fabiola Giudici, Francesca Boscolo Nata, Alberto Marcuzzo, Paolo Boscolo-Rizzo

**Affiliations:** 1 Department of Medical, Surgical and Health Sciences, Section of Otolaryngology, University of Trieste, Strada Di Fiume, 447, 34149 Trieste, Italy; 2 Unit of Cancer Epidemiology, Centro di Riferimento Oncologico di Aviano (CRO), Istituto di Ricovero e Cura a Carattere Scientifico (IRCCS), Aviano, Italy; 3 Department of Medical, Surgical and Health Sciences, Section of Plastic Surgery, University of Trieste, Trieste, Italy; 4 Department of Life Sciences, University of Trieste, Trieste, Italy

**Keywords:** Oral cavity cancer, Squamous cell carcinoma, Narrow band imaging, Surgery, Margin mapping, Survival

## Abstract

**Purpose:**

The study aimed to assess if outcomes for oral squamous cell carcinoma (OSCC) patients have improved, and if so, whether these improvements correlate with changes in diagnostic and therapeutic methods over time.

**Methods:**

Retrospective study including patients surgically treated for OSCC between 2002 and 2020.

**Results:**

Among the 193 consecutive patients with primary OSCC who met the inclusion criteria (median age 66; 60.4% male), 80 (41.4%) were treated between 2002 and 2011, and 113 (58.6%) between 2011 and 2020. Multivariate analysis showed a significant improvement in overall survival rates from 2012 to 2020, compared with the period from 2002 to 2011 (HR for death, 0.33; 95% CI 0.17–0.67). Similar observations have emerged in progression-free and disease-specific survival. When stratified by stage, the improvement was found to be significant only for advanced stages. The use of NBI during both preoperative and operative setting as well as margin mapping significantly increased over the time. Both patients with early and advanced-stage OSCC treated between 2012 and 2020 exhibited improved pain scores.

**Conclusions:**

Despite the shift to less invasive surgeries, our analysis shows significant improvement over time, especially in advanced-stage diseases, highlighting the impact of evolving strategies on challenging cases.

## Introduction

The management and treatment of oral squamous cell carcinoma (OSCC) have been an evolving landscape marked by innovations, advancements, and methodological refinements over the decades [1]. In recent years, significant efforts have been made to reduce the invasiveness of surgical interventions, with the dual goal of maintaining, if not improving, survival rates while significantly enhancing the quality of life for patients [2]. With this in mind, there has been a significant shift in therapeutic strategies from radical surgeries to more conservative approaches that prioritize quality of life while still achieving effective therapeutic outcomes. [3].

In this landscape, the introduction of technologies like narrow band imaging (NBI) and practices such as margin mapping have emerged as pivotal and innovative elements [4]. NBI has proved helpful in the early detection of primary OSCC and recurrences and in delineating superficial tumor boundaries more accurately during surgeries. [5–7]. Margin mapping, on the other hand, aims to ensure complete tumor resection by methodically examining intraoperatively the surgical margins [8]. In this way, a more conservative and tailored resection can be performed, going beyond the traditional rule of maintaining a macroscopic surgical margin of at least 1.5–2 cm. [9].

The surgical techniques themselves have also undergone transformation. The advent of transoral surgery also for advanced-stage diseases heralded a new era in the surgical management of OSCC [10]. This less invasive technique offers significant advantages, including a reduced reliance on the use of free flaps, shorter hospital stays, and, importantly, faster return to oral intake and speech functions [11]. Furthermore, the reliance on and emphasis given to compartmental surgery of the oral cavity has been scaled back in recent years [12]

Despite the above recent technological and therapeutic advancements in the management and treatment of OSCC, it remains unclear whether these innovations have translated into improved survival rates compared to traditional approaches used in previous decades. This observation induced our institute to conduct an evaluation regarding the evolution of survival rates for OSCC at our center over the past decades.

## Materials and Methods

### Study Population and Data

This study was designed as a retrospective cohort analysis of patients diagnosed and treated for OSCC between January 2002 and November 2020 at Trieste University Hospital, a tertiary referral center. The study protocol was approved by the University of Trieste Ethics Committee on Clinical Investigation (N.89/2018) in compliance with the Helsinki Declaration. Patients were included in the study if they met all of the following criteria: adults of both sexes aged 18 years or older, histopathologically confirmed diagnosis of non-metastatic (M0) OSCC, underwent upfront surgical treatment with curative intent between January 2002 and November 2020, comprehensive medical records and follow-up data available, no evidence of distant metastases or other synchronous head and neck cancers at diagnosis, no previous treatment for head and neck cancer, and no neoadjuvant therapy administered prior to surgery. The study excluded patients with distant metastases and/or synchronous head and neck squamous cell carcinoma, unavailable follow-up information, previous treatments for head and neck cancer, other synchronous or metachronous primary tumors, or treatment with neoadjuvant therapy. Upfront surgery was performed with curative intent by the same surgeon (GT), who initially had a robust 15-year background in head and neck oncology. Resection was planned according to the tumor subsite and extension. Selective or modified radical neck dissections were performed in adherence with the National Comprehensive Cancer Network (NCCN) guidelines [13]. Over the years, en bloc resection was employed only in cases where there was continuity between the primary tumor and the nodal disease [14]. Adjuvant treatment was also in adherence with NCCN guidelines.

### Margin Mapping

We performed margin mapping by collecting frozen sections (FS) from the surgical bed using a defect-driven approach. These sections were obtained as 3-to-4-mm-thick strips of tissue surrounding the tumor for superficial margins and as a bowl of tissue beneath it for the deep margins. While during the surgical excision, instruments such as vessel sealing and dissection devices were employed, for margin mapping, CO_2_ waveguide laser was utilized to minimize thermal damage [4, 7, 15] and increase accuracy by reducing artifacts on specimens. Following the protocol outlined by Hinni et al. [8], the surgeon applied ink to the most lateral surface of each FS before presenting them to the pathologist.

### Variables

Clinical and pathological data were collected from patient records, encompassing demographics, surgical techniques for primary tumor removal, and comprehensive pathologic findings such as margin status, tumor grade, depth of invasion (DOI), extracapsular extension (ECE), and pathologic stage. In cases where specific pathological characteristics were not documented in the clinical records, the specimens were re-evaluated by a pathologist who then provided the missing information. All cases were classified or reclassified based on the latest AJCC-TNM 8th edition staging criteria [16]. The study also documented whether each case underwent multidisciplinary cancer conference (MCC), and the application of NBI in diagnosis, tumor margin mapping, and follow-up. Additionally, the use of intraoperative FS analysis for immediate pathologic evaluation was noted. The duration of hospital stay and the occurrence of any postoperative complications were also documented.

### Follow-UP

Each patient was systematically monitored through a routinary follow-up, which included an endoscopic fiber optic evaluation of the upper aero-digestive tract also using NBI [17, 18]. Follow-up evaluations were scheduled as follows: every one to three months during the first year, every three to four months in the second year, every four to six months in the third year, and semiannually thereafter. Subsequently, patients were assessed annually, in accordance with consensus guidelines.

### Post-Treatment QoL Assessment

Six months after the end of treatment, the QoL was assessed using the version 4 of the University of Washington Quality of Life Questionnaire (UW-QOL v4) [19].

### Statistical Methods

Sociodemographic and clinical characteristics were reported as a percentage, and differences between anterior and posterior localization were compared through Fisher’s exact test. A survival analysis was conducted to evaluate the association between sociodemographic characteristics and oncological outcomes, namely progression-free survival (PFS), overall survival (OS), and disease-specific survival (DSS). For each patient, the person-time at risk was calculated between the date of surgery and the event date or the last follow-up, whichever occurred first. The event of interest was defined as recurrence/progression (any site) or death for PFS, death for OS, and cancer-specific death for DSS. Survival probabilities for PFS, OS, and DSS were estimated using the Kaplan–Meier method, and differences across strata were evaluated using the log-rank test [20]. The risk of unfavorable oncological outcomes was evaluated using the Cox proportional hazards model; multivariable hazard ratios (HR), and corresponding 95% confidence intervals (CI), were calculated [20]. Analyses were performed using *R* 3.6. and statistical significance was claimed for *p* < 0.05 (two-tailed).

## Results

A total of 193 consecutive patients with primary OSCC (median [range] age, 66 [35–89] years; 117 [60.4%] males) met inclusion criteria and were analyzed (Table 1). The patients were divided into two groups to assess changes in outcome between 2002 and 2011 (*n* = 80 patients; 41.4%) and between 2012 and 2020 (*n* = 113 patients; 58.6%).

**Table 1:** Socio-demographic and clinical characteristics according to period of surgery

	All patients		Period of surgery				
			2002–2011		2012–2020		Fisher’s exact test
	*n*	(%)	*n*	(%)	*n*	(%)	
*Gender*							
Female	76	(39.4)	29	(36.3)	47	(41.6)	*p* = 0.550
Male	117	(60.6)	51	(63.8)	66	(58.4)	
*Age (years)*							
Median		66		60		68	*p* = 0.001^a^
(Min–Max)		(35–89)		(35–84)		(35–89)	
*Surgical margins*							
Free	171	(88.6)	71	(88.8)	100	(88.5)	*p* = 1.000
Close/Inf	22	(11.4)	9	(11.3)	13	(11.5)	
*Extracapsular extension*							
No	172	(89.1)	65	(81.3)	107	(94.7)	*p* = 0.004
Yes	21	(10.9)	15	(18.8)	6	(5.3)	
*pT*							
pT1	57	(29.5)	21	(26.3)	36	(31.9)	*p* = 0.509
pT2	52	(26.9)	19	(23.8)	33	(29.2)	
pT3	50	(25.9)	24	(30.0)	26	(23.0)	
pT4	34	(17.6)	16	(20.0)	18	(15.9)	
*pN*							
pN0	134	(69.4)	47	(58.8)	87	(77.0)	*p* = 0.011
pN1-pN3	59	(30.6)	33	(41.3)	26	(23.0)	
*pStage*							
Early (I-II)	94	(48.7)	32	(40.0)	62	(54.9)	*p* = 0.015
Late (III-IVb)	99	(51.3)	48	(60.0)	51	(45.1)	
DOI (mm)							
< 5	100	(51.8)	22	(27.5)	25	(22.1)	*p* = 0.161
5 to 10	47	(24.4)	35	(43.8)	65	(57.5)	
> 10	46	(23.8)	23	(28.8)	23	(20.4)	
*Preoperative NBI*							
Yes	82	(42.5)	0	(0.0)	82	(72.6)	*p* < 0.001
No	111	(57.5)	80	(100)	31	(27.4)	
*Frozen*							
Yes	142	(73.6)	50	(62.5)	92	(81.4)	*p* = 0.005
No	51	(26.4)	30	(37.5)	21	(18.6)	
*Margins mapping by means of strip-and-bowl*							
Yes	124	(64.3)	41	(51.3)	83	(73.5)	*p* = 0.002
No	69	(35.8)	39	(48.7)	30	(26.6)	
*NBI-guided margins definition*							
Yes	52	(26.9)	1	(1.3)	51	(45.1)	*p* < 0.001
No	141	(73.1)	79	(98.8)	62	(54.9)	

	All patients		Period of surgery				
			2002–2011		2012–2020		Fisher’s exact test
	*n*	(%)	*n*	(%)	*n*	(%)	
*MCC*							
Yes	114	(59.1)	1	(1.3)	113	(100)	*p* < 0.001
No	79	(40.9)	79	(98.8)	0	(0.0)	
*CO*_*2*_* waveguide laser*							
Yes	54	(28.0)	0	(0.0)	54	(47.8)	*p* < 0.001
No	139	(72.0)	80	(100)	59	(52.2)	
*Open surgery*							
No	130	(67.4)	48	(60.0)	82	(72.6)	*p* = 0.086
Yes	63	(32.6)	32	(40.0)	31	(27.4)	
*En bloc resection*							
No	121	(62.7)	39	(48.8)	82	(72.6)	*p* < 0.001
Yes	72	(37.3)	41	(51.3)	31	(27.4)	
*Flap*							
No	109	(56.5)	49	(61.3)	60	(53.1)	*p* = 0.423
Regional flap	42	(21.8)	14	(17.5)	28	(24.8)	
Free flap	42	(21.8)	17	(21.3)	25	(22.1)	
Anterolateral thigh	16	(38.1)					
Radial forearm	12	(28.6)					
Fibular	10	(23.8)					
Ulnar forearm	4	(9.5)					
*Complications*							
No	147	(76.2)	57	(71.3)	90	(79.7)	*p* = 0.230
Yes	46	(23.8)	23	(28.8)	23	(20.4)	
*Duration of hospitalization (days)*							
≤ 10	72	(37.3)	29	(36.3)	43	(38.1)	*p* = 0.032
11–20	80	(41.5)	27	(33.8)	53	(46.0)	
≥ 21	41	(21.2)	24	(30.0)	17	(15.0)	

^a^Mann–Whitney test

### Differences in Demographic and Clinical Characteristics Between the two Groups

Differences in demographic and clinical characteristics between the two groups are reported in Table 1. There was a significant reduction in ECE from 18.8% in the earlier period to 5.3% in the later period (*p* = 0.004). The distribution of tumor stages (*p* = 0.015) and nodal involvement (*p* = 0.011) showed a significant shift toward earlier stages and less nodal involvement in the later period. There was a notable increase in the preoperative use of NBI from 0% in the earlier period to 72.6% in the later period (*p* < 0.001). Similarly, the use of FS increased significantly (*p* = 0.005). The practice of margins mapping increased over time, with 73.5% in the later period compared to 51.3% in the earlier period (*p* = 0.002). NBI-guided superficial surgical margins definition, the use of CO_2_ waveguide laser as well as MCC-based treatment planning approach also increased dramatically (*p* < 0.001). A notable trend in surgical approaches for oral cancer treatment over the two periods studied was observed with open surgery (*p* = 0.086) and en bloc resection (*p* < 0.001) being used less common in the more recent period.

### Outcome of Patients in Each Group

**Fig. 1:**
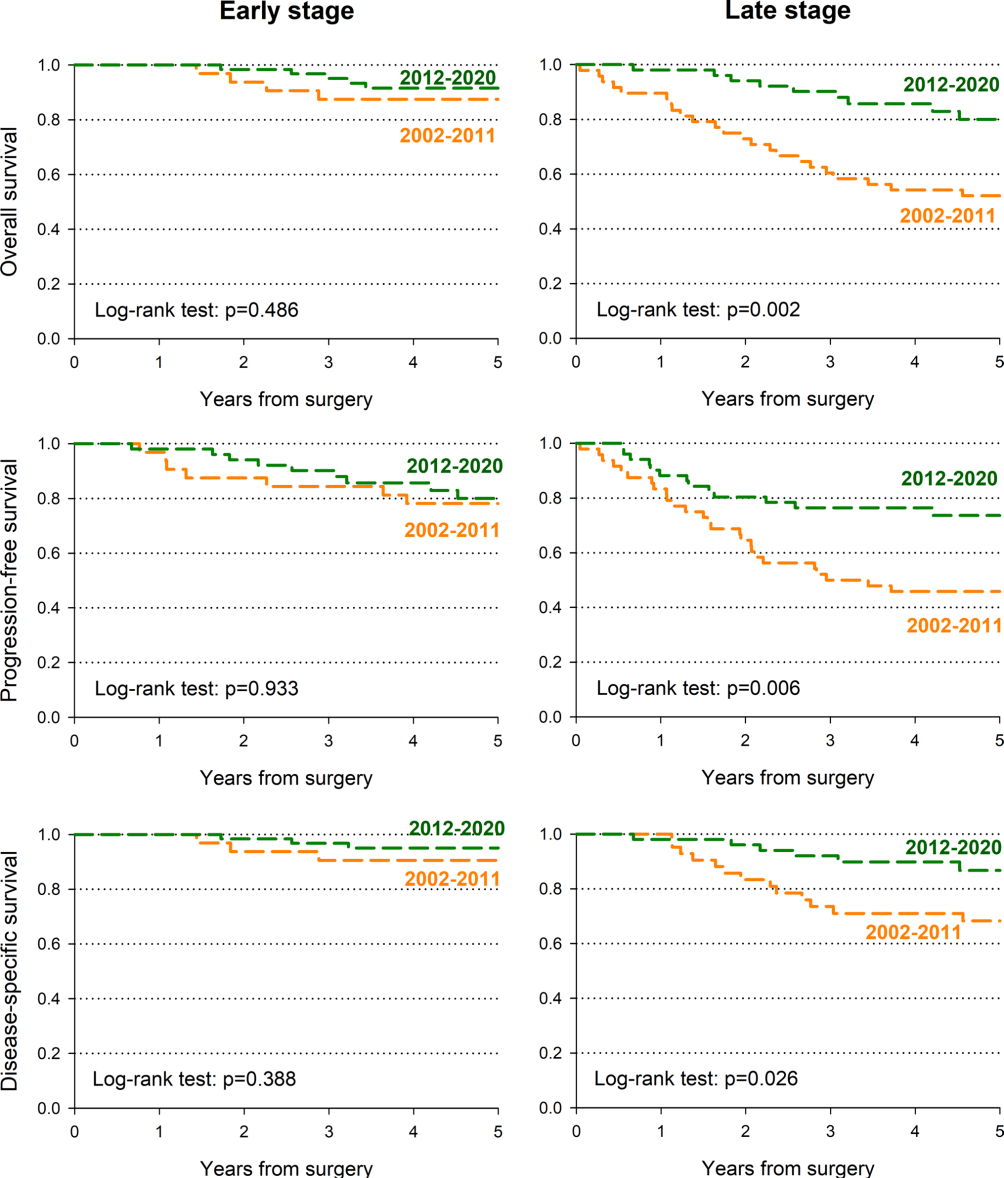
Kaplan–Meier estimates of overall, progression-free, and disease-specific survival according to cancer stage and period of surgery

Significant differences were observed in hospitalization duration between groups (*p* = 0.032). Patients treated in the earlier period had higher rates of longer stays (≥ 21 days: 30.0%) compared to those treated in the later period (15.0%). Complication rates did not differ significantly between groups (Table 1). At multivariate analysis, it was observed that from 2012 to 2020, the OS rates showed a significant improvement compared to the period from 2002 to 2011 (HR for death, 0.33; 95% CI 0.17–0.67) (Table 2). This improvement was particularly noteworthy in advanced-stage patients (HR: 0.27; 95% CI 0.12–0.63), but not statistically significant in early-stage cases (HR: 0.53; 95% CI 0.14–1.99). Similarly, from 2012 to 2020, patients experienced an improvement in PFS (HR: 0.54; 95% CI 0.31–0.93) compared to the period from 2002 to 2011. This improvement was significant in patients with advanced-stage disease (HR: 0.35; 95% CI 0.17–0.72). DSS followed this pattern too, showing an improvement in the later period for all patients (HR: 0.39; 95% CI 0.16–0.93) but lacking statistical significance when stratifying for stage. Kaplan–Meier estimates of OS and PFS according to cancer stage and period of surgery are reported in Fig. 1 Given the study sample size of 80 and 113 patient in the two study periods, a post hoc power analyses on the HR of death between the two considered periods revealed a power ≥ 80% for HR ≤ 0.41, given an overall survival rate of 70% and fixing *α* = 0.05.

**Table 2:** Hazard ratio (HR) of death and progression/death within 5 years from surgery with corresponding 95% confidence interval (CI) according to stage and calendar period of surgery

Calendar period of surgery	All patients		Early stage		Advanced stage	
	*n*	HR (95% CI)^a^	*n*	HR (95% CI)^b^	*n*	HR (95% CI)^b^
*Overall survival*						
2002–2011	80	Ref	32	Ref	48	Ref
2012–2020	113	0.33 (0.17–0.67)	64	0.53 (0.14–1.99)	49	0.27 (0.12–0.63)
*Progression-free survival*						
2006–2012	80	Ref	32	Ref	48	Ref
2013–2020	113	0.54 (0.31–0.93)	64	0.91 (0.36–2.30)	49	0.35 (0.17–0.72)
*Disease-specific survival*						
2006–2012	80	Ref	32	Ref	48	Ref
2013–2020	113	0.39 (0.16–0.93)	64	0.47 (0.09–2.36)	49	0.39 (0.13–1.17)

^a^Estimated from Cox proportional hazards model adjusting for gender, age, stage, surgical margins, and extracapsular extension

^b^Estimated from Cox proportional hazards model adjusting for gender, age, surgical margins, and extracapsular extension

### Quality of Life

Patients treated in the first period revealed significantly lower QOL scores for pain with mean score increasing from 85.9 to 95.3 (*p* = 0.001) (Table 3). Significant improvements were noted in the domains of speech and taste. Speech scores improved from 83.3 to 91.6 (*p* = 0.008), and taste scores improved from 83.1 to 92.1 (*p* = 0.008). For the other domains like appearance, though there were changes in scores, these did not reach statistical significance. The composite score showed a trend toward improvement (from 56.5 to 61.5), but this was not statistically significant (*p* = 0.124). When stratified by stage, both patients with early and advanced-stage OSCC treated between 2012 and 2020 exhibited improved pain scores (Table 3).

**Table 3:** Socio-demographic and clinical characteristics according to period of surgery

Domain	All patients			Early stage			Advanced stage		
	2002–2011	2012–2020	Student’s *t* test	2002–2011	2012–2020	Student’s *t* test	2002–2011	2012–2020	Student’s *t* test
	Mean (Sd)	Mean (Sd)		Mean (Sd)	Mean (Sd)		Mean (Sd)	Mean (Sd)	
Pain	85.9 (21.8)	95.3 (13.4)	*p* = 0.001	91.8 (15.4)	99.2 (4.4)	*p* = 0.010	77.1 (26.7)	92.4 (16.8)	*p* = 0.003
Appearance	86.0 (16.0)	87.1 (17.0)	*p* = 0.683	89.8 (14.0)	90.9 (14.9)	*p* = 0.755	83.1 (17.0)	81.4 (18.5)	*p* = 0.672
Activity	88.7 (21.9)	90.8 (17.9)	*p* = 0.495	90.6 (20.8)	93.3 (15.7)	*p* = 0.511	87.2 (22.7)	87.1 (20.5)	*p* = 0.989
Recreation	89.7 (20.6)	82.2 (16.3)	*p* = 0.376	93.8 (18.0)	95.2 (9.9)	*p* = 0.637	86.6 (22.1)	87.9 (22.2)	*p* = 0.808
Swallowing	77.7 (28.8)	84.3 (23.5)	*p* = 0.108	85.6 (26.8)	89.1 (18.9)	*p* = 0.484	71.7 (29.2)	77.1 (27.7)	*p* = 0.409
Chewing	67.7(37.2)	74.4 (28.0)	*p* = 0.190	79.7 (32.7)	83.2 (25.1)	*p* = 0.584	58.7 (38.1)	61.4 (27.3)	*p* = 0.726
Speech	83.3 (24.2)	91.6 (14.7)	*p* = 0.008	86.3 (20.3)	92.5 (13.1)	*p* = 0.090	81.2 (26.7)	90.3 (16.9)	*p* = 0.084
Taste	83.1 (27.8)	92.1 (13.3)	*p* = 0.009	89.1 (19.9)	94.2 (11.9)	*p* = 0.140	78.6 (32.0)	88.9 (14.7)	*p* = 0.084
Shoulder	86.5 (23.0)	88.4 (24.0)	*p* = 0.617	90.0 (19.7)	89.6 (23.2)	*p* = 0.938	84.0 (25.1)	86.6 (25.3)	*p* = 0.649
Saliva	80.7 (30.5)	87.5 (21.2)	*p* = 0.097	90.7 (21.7)	92.7 (16.7)	*p* = 0.625	73.3 (34.0)	79.7 (24.8)	*p* = 0.351
Mood	80.7 (22.0)	83.3 (19.7)	*p* = 0.417	85.2 (20.9)	89.9 (15.9)	*p* = 0.242	77.3 (22.4)	73.6 (21.0)	*p* = 0.451
Anxiety	74.4 (32.8)	81.5 (27.4)	*p* = 0.136	78.8 (30.2)	89.4 (19.4)	*p* = 0.054	71.2 (34.7)	69.7 (33.1)	*p* = 0.852
Composite score	56.5 (21.4)	61.5 (19.6)	*p* = 0.124	63.1 (19.0)	68.0 (16.6)	*p* = 0.225	51.6 (22.0)	51.6 (19.9)	*p* = 0.996

## Discussion

This investigation reveals a notable increase in survival rates over time. Being treated in the last decade has proven to be an independently favorable factor, as shown by multivariate analysis. Notably, the recent period saw more early-stage cases treated, but a stage-stratified analysis indicates significant benefits for advanced-stage patients. In early-stage OSCCs, surgical resection is more straightforward, often easily achievable with a 1.5-cm margin from the tumor. Consequently, the benefits of technological advancements, significant in enhancing survival rates in advanced stages, might have a comparatively modest effect in this particular subgroup.

While other researches have demonstrated improved survival rates in oral cavity cancer in the last decade, these findings predominantly stem from multi-institutional cancer registry data [21–23]. Such large-scale studies, while valuable, often face challenges like heterogeneity of data sources, limited access to detailed clinical information, and variability in patient follow-up procedures. Our mono-institutional study addresses these issues by providing a more controlled, detailed, and consistent dataset, allowing for directly associate the observed improvements in survival with the implementation of advanced diagnostic and therapeutic techniques specific to our institution. The same surgeon and team of pathologists and radiologists managed patients throughout our series, ensuring consistency in surgical approach and diagnostic and postoperative evaluations. Furthermore, the follow-up strategies employed were stringent and uniform across all cases, ensuring a consistent monitoring approach.

The observation that the improvement in survival rates among patients with OSCC has been linked to increased use of innovative diagnostic techniques is noteworthy. Techniques like NBI have been increasingly utilized not only in the diagnostic phase, but also during surgical procedures. The use of FS has also become recently more common. Additionally, the MCC discussion of clinical cases, involving experts from various fields, has become a vital part of the treatment process. While these advancements are associated with better survival outcomes, it is important to clarify that this is an association rather than a direct cause-and-effect relationship. The complexity of cancer treatment means that many factors contribute to patient outcomes, and while these innovative techniques are certainly beneficial, they are part of a broader array of factors influencing patient survival.

NBI usage potentially improves diagnostic accuracy before treatment and defines surgical margins more precisely during operations [5, 24], possibly leading to more effective cancer removal while preserving vital healthy tissue, crucial for patient recovery and reducing recurrence [7].

Additionally, the use of FS may have significantly contributed to accurately assessing surgical excision completeness. Its real-time feedback allows surgeons to adjust removal during the procedure, potentially achieving complete tumor removal in one surgery. Recent trends, such as the ‘defect-driven margin mapping’ with the ‘strip-and-bowl’ technique, further enhance margin assessment and tumor removal precision [15]. This approach not only improves surgical precision but also reduces the need for additional surgeries, potentially improving patient outcomes [21].

Also, we noted a significant trend in the use of the CO_2_ waveguide laser, exclusively observed in recent periods. This preference is due to its superior efficacy compared to other non-conventional cutting tools, showing reduced thermal damage and improved FS readability [18, 25]. These factors may improve surgical radicality and define surgical margins more accurately [25]. Additionally, the laser’s distinct interaction with healthy mucosa or cancer aids in determining tumor depth extension [8, 18].

The increase in the discussion of clinical cases within MCC in the recent period is significant and may have contributed to improved survival rates. A recent meta-analysis has shown that patients managed by MCCs exhibited increased OS compared to control patients [26], indicating that a collaborative approach among diverse specialists positively impacts patient outcomes.

It is also noteworthy that such improvements have occurred despite a reduced reliance on open surgery and en bloc resection. While open surgery and en bloc resection have been traditional methods, their associated morbidity and impact on quality of life have prompted a shift toward more conservative approaches. These methods can help achieve adequate tumor removal while preserving as much healthy tissue as possible, reducing the need for complex free flap reconstructions.

In particular, some authors advocate the en bloc approach for treating T2-T3 cancers, citing the presence of the T-N tract as justification. This tract is defined as all anatomical structures located between the tongue (*T*) and the first nodal level (*N*). It encompasses the sublingual and submandibular glands, the mylohyoid muscles, the lingual arteries, veins, and nerves, the hypoglossal nerves, the Wharton ducts, the lingual and sublingual nodes, and stromal tissue. [27]. In our recent clinical experience, the decisive factor in approach selection has been the invasion of the mylohyoid muscle, serving as a vital barrier between the oral cavity and neck compartments. When MRI confirms muscle invasion, we opt for compartmental surgery and utilize a free flap. On the other hand, if MRI does not definitively indicate muscle invasion, an intraoperative FS assessing potential infiltration up to the mylohyoid muscle allows us to convert the transoral approach to a compartmental approach. [25]

The analysis of QoL revealed several more favorable scores in patients treated in the most recent period, even after stratifying by stage. Specifically, pain scores six months post-treatment were more favorable in the recent group. This suggests that changes in diagnostic and therapeutic strategies may have positively impacted not only prognosis, but also this aspect of QoL. However, recent trends indicate rising expectations and demands from head and neck cancer patients [28], potentially obscuring the true extent of QoL improvements across different patient groups.

The present study has several limitations that warrant attention. First and foremost, its retrospective cohort design inherently imposes limitations, as it relies on historical data that may lack certain relevant information and are subject to selection bias. Additionally, while our single-center study allowed for a controlled and consistent dataset, future multicenter studies could provide a broader perspective, larger sample size, and increased generalizability of the findings across different healthcare settings and populations. Also, the follow-up period, though extended, might not be sufficient to fully assess long-term outcomes, particularly for QoL, which can vary significantly over time. Additionally, the QoL assessment was only conducted six months post-treatment, a time frame that might not fully reflect the patients’ experiences throughout their recovery and rehabilitation. Lastly, it is crucial to consider that the study’s conclusions are based on associative observations and do not provide direct evidence of causality. While technological advancements and multidisciplinary treatment strategies may be associated with improved outcomes, other unassessed factors in the study could significantly impact patient outcomes. This underscores the need for further research to better understand the impact of these evolving strategies and technologies on oral cancer prognosis.

## Conclusions

The present investigation suggests that the observed improvement in survival outcomes for advanced-stage disease patients might be attributed to recent advancements in diagnostic and treatment techniques, such as the growing implementation of NBI during the diagnostic phase, the employment of margin mapping combined with NBI technique, the utilization of CO_2_ waveguide laser technology, and the application of FS analysis during surgical procedures, despite the observed trend toward less invasive surgical approaches. Additionally, the shift toward more multidisciplinary and collaborative approaches in patient care could also be playing a role. However, these are associative observations and further studies are necessary to confirm these hypotheses and fully understand the impact of these evolving strategies and technologies in OSCC prognosis.

## Data Availability

The authors confirm that the data supporting the findings of this study are available within the article.
